# Mechanisms of Fibroblast Activation and Myocardial Fibrosis: Lessons Learned from FB-Specific Conditional Mouse Models

**DOI:** 10.3390/cells10092412

**Published:** 2021-09-14

**Authors:** Prachi Umbarkar, Suma Ejantkar, Sultan Tousif, Hind Lal

**Affiliations:** 1 Division of Cardiovascular Disease, The University of Alabama at Birmingham, Birmingham, AL 35294, USA; tsultan@uabmc.edu; 2 School of Health Professions, The University of Alabama at Birmingham, Birmingham, AL 35294, USA; sumae323@uab.edu

**Keywords:** fibrosis, fibroblast, TGF-β, GSK-3, GRK, p38

## Abstract

Heart failure (HF) is a leading cause of morbidity and mortality across the world. Cardiac fibrosis is associated with HF progression. Fibrosis is characterized by the excessive accumulation of extracellular matrix components. This is a physiological response to tissue injury. However, uncontrolled fibrosis leads to adverse cardiac remodeling and contributes significantly to cardiac dysfunction. Fibroblasts (FBs) are the primary drivers of myocardial fibrosis. However, until recently, FBs were thought to play a secondary role in cardiac pathophysiology. This review article will present the evolving story of fibroblast biology and fibrosis in cardiac diseases, emphasizing their recent shift from a supporting to a leading role in our understanding of the pathogenesis of cardiac diseases. Indeed, this story only became possible because of the emergence of FB-specific mouse models. This study includes an update on the advancements in the generation of FB-specific mouse models. Regarding the underlying mechanisms of myocardial fibrosis, we will focus on the pathways that have been validated using FB-specific, in vivo mouse models. These pathways include the TGF-β/SMAD3, p38 MAPK, Wnt/β-Catenin, G-protein-coupled receptor kinase (GRK), and Hippo signaling. A better understanding of the mechanisms underlying fibroblast activation and fibrosis may provide a novel therapeutic target for the management of adverse fibrotic remodeling in the diseased heart.

## 1. Introduction 

Fibrosis is characterized by the excessive accumulation of extra cellular matrix (ECM) components. It is a physiological response to pathological stimuli that helps to confine injuries. However, the prolonged activation of this process results in adverse tissue remodeling, which can ultimately affect the structure and function of organs (adverse remodeling). Fibroblasts (FBs) are the major contributor to fibrosis. Previous studies report that the epicardium, endothelial cells, bone-marrow-derived cells, and perivascular cells could be the origins of activated FBs [1,2,3,4,5,6,7,8]. However, recent genetic lineage tracing experiments have confirmed that most activated FBs in the heart originate from tissue-resident FBs [9,10,11,12]. In a normal heart, FBs are quiescent/resting/non-activated. However, in the injured heart, FBs go through a continuum of transient intermediary states and contribute significantly to the cardiac remodeling process [13,14,15,16]. For instance, when myocardial infarction (MI) occurs, a range of stimuli activate resting FBs, giving rise to a new cell state known as activated FBs/myofibroblast. Activated FBs are pro-inflammatory, hyper-secretory, and hyper-migratory in nature. As the healing phase progresses towards scar formation, activated FBs acquire anti-inflammatory and pro-angiogenic phenotypes. They secrete cytokines, ECM components, and other necessary paracrine factors that are required for wound healing. After scar maturation, FBs regress to the quiescent stage or acquire a specialized phenotype (matrifibrocyte) and remain in the matured scar [14,16]. A better understanding of the mechanisms underlying fibroblast activation and fibrosis may provide a novel therapeutic target for managing adverse fibrotic remodeling in the diseased heart. 

## 2. Studies of FB-Specific In Vivo Mouse Models 

Recent advancements in the generation of FB-specific mouse models have evolved the field of fibroblast biology. Table 1 lists proteins/markers that have been targeted to create numerous mouse lines for FB-specific genetic manipulation and lineage tracing. The major limitation associated with these models is non-specificity, as many of these markers are expressed by cells other than FBs such as pericytes, vascular smooth muscle cells (VSMC), endothelial cells, immune cells, and cardiomyocytes or show transient expression in FBs. Several studies have employed fibroblast-specific protein 1 (*FSp1*)-*Cre* and *Col1a2-Cre* for fibroblast-specific gene targeting [6,17]. However, in recent years, a growing body of evidence has challenged the specificity of FSP1 and COL1A2 as fibroblast markers [18,19]. Kong et al. [18] reported that the majority of FSP1+ cells in the infarcted myocardium are identified as hematopoietic or endothelial cells; therefore, FSP1 is not a specific marker for FBs in the heart. Consistently, in the pressure overload model, FSP1 was primarily expressed by hematopoietic and vascular cells [18]. Multiple studies have raised similar concerns regarding the specificity of *Col1a2-Cre* to fibroblasts, which also express in many other parenchymal cell types, including epicardial cells and valvular interstitial cells [9,12,20]. Taken together, multiple studies have raised significant concerns about the specificity of FSP1 and COL1A2 as fibroblast markers. Due to these limitations, *FSp1*- or *Col1a2*-driven gene deletion is of limited value in the dissection of the role of FBs in the pathophysiology of cardiac diseases [18].

Presently, a growing consensus supports the notion that inducible transcription factor 21 (*Tcf21*)-*MerCreMer* (*MCM*) and periostin (*Postn*)-*MCM* mouse lines are the most reliable tools for fibroblast- or myofibroblast-specific gene targeting. Indeed, both the *Postn-MCM* and *Tcf21-MCM* are inducible; hence, gene deletion will only start once mice are subjected to Tamoxifen. Therefore, these new models avoid any unwanted confounding developmental effects. Periostin is a matricellular protein secreted by FBs. Periostin expression is negligible in the normal heart, while in the injured myocardium, periostin starts expressing in the FB population. Molkentin et al. [9] created a knock-in mouse (*Postn-MCM*), in which tamoxifen-inducible Cre recombinase expression cassette was inserted into the *Postn* gene locus. Their elegant report demonstrated that the *Postn-MCM* system could trace all the activated FBs/myofibroblasts in the injured heart, thus making this mouse line a valuable tool for myofibroblast-specific gene targeting. In addition to this targeted periostin knock-in model, a transgenic *Postn-CreER2* was reported as well [21]. The key difference between the transgenic Cre driven by a fragment of the periostin promoter and knocking the Cre cassette into the periostin locus is that the expression of the Cre in the knock-in model represents the true endogenous regulation of periostin gene expression; whereas, the periostin-promoter-driven transgenic Cre may not have a complete set of regulatory information for that locus and thus does not fully represent the endogenous regulation. Since periostin expression is negligible-to-none in quiescent FBs, these animals are of limited value for FB-specific gene targeting in the healthy heart. This hurdle is overcome by the inducible *Tcf21-MCM* mouse model. TCF21 is a member of the bHLH (basic helix–loop–helix) family of transcription factors. It is expressed extensively during embryogenesis and it is essential to the development of cardiac FBs [20]. Importantly, in a healthy heart, a large number of resident fibroblasts are found to be TCF21+. Moreover, lineage-tracing experiments confirmed that TCF21+ cells are the primary source of myofibroblasts in the injured heart [9]. Taken together, these features enable the *Tcf21-MCM* system to target genes before cardiac injury and in all transient forms of FBs post-injury. 

As noted above, traditionally, most of the research on fibroblast activation and fibrosis was centered on the canonical TGF-β signaling pathway [81,82,83]. However, the recent emergence of these conditional FB-specific mouse models has facilitated the identification of several additional novel pathways that are critical to fibroblast activation and fibrosis. Studies carried out with FB-specific, genetically manipulated mouse models are listed in Table 2. These pathways may operate independently or in co-ordination with TGF-β signaling. On the other hand, these updated tools have helped to redefine several hypotheses and concepts that exclusively relied on isolated culture models or mouse models that were later identified as non-specific. These included the views regarding the sources of FBs in the failing heart and the ability of activated fibroblasts to revert to the quiescent stage. Specifically, in complete disagreement with traditional beliefs, the new genetic models identified that most activated fibroblasts in the failing heart originate from existing resident fibroblasts, and activated fibroblasts can revert to the quiescent stage, once the stress is released [9,14]. These findings have enormous implications for human health; for example, the latter condition is directly comparable to that of patients with ventricular assistance devices (VAD). Furthermore, the discovery of fibroblast’s ability to revert to the quiescent stage opens the door to the potential reverse remodeling of fibrotic hearts.

In summary, the field of cardiac fibroblast and fibrosis research is going through a transition process. Traditional beliefs are being challenged, and novel pathways are being identified, leading to new and emerging concepts. This revolutionary transition is primarily driven by the emergence of newly developed, inducible, FB-specific mouse models. In the following sub-sections of this review article, we will specifically focus on the mechanisms of fibrosis that have been validated by multiple studies with FB-specific, in vivo mouse models. 

### 2.1. TGF-β1 Signaling Pathway in Myocardial Fibrosis

The transforming growth factor-β (TGF-β) is a superfamily of 30 ligands that belongs to three main subfamilies: (1) TGF-β; (2) activins/inhibins/Nodal; and (3) bone morphogenetic proteins (BMPs). Among these, the TGF-β pathway has been considered a key mediator of fibroblast activation and fibrosis in the diseased heart [81,85]. There are three isoforms of TGF-β ligands: TGF-β1, TGF-β2, and TGF-β3. Despite having a remarkable homology, these ligands demonstrate distinct biological functions. In the canonical TGF-β signaling pathway, TGF-β ligands bind to Type II serine/threonine kinase receptors, which further trans-phosphorylate the Type I receptor’s kinase domain. These receptor kinases activate receptor-associated SMADs (R-SMADs), specifically SMAD2/3. Activated SMADs form a heteromeric complex with Co-SMAD, i.e., SMAD4. This complex translocates into the nucleus, binds to SMAD binding elements (SBE), and regulates the transcription of target genes. TGF-β signaling is negatively regulated by inhibitory SMADs (I-SMADs), namely SMAD6 and SMAD7. I-SMADs competitively inhibit R-SMADs activation at the Type I receptor or prevent the formation of the effector R-SMAD-Co-SMAD complex. Furthermore, I-SMADs are involved in the ubiquitination and degradation of Type I receptors [86,87]. In addition to the classical SMAD-dependent (canonical) pathway described above, TGF-βs act in a SMAD-independent manner (non-canonical) through non-canonical mediators, such as TGF-β-activated kinase 1 (TAK1), tumor necrosis factor (TNF), MAP kinases (ERK, p38, and JNK), Rho kinase, phosphoinositide 3-kinase (PI3K), AKT, and nuclear factor-κB (NF-κB) [88,89,90,91,92].

In order to investigate the fibroblast-specific role of canonical TGF-β signaling in the diseased heart, Khalil et al. [33] selectively deleted *Tgfbr1/2*, *Smad2*, or *Smad3* using fibroblast specific Cre drivers. These authors observed that the fibroblast-specific double deletion of *Tgfbr1/2* or *Smad2/3* protected from TAC-induced myocardial fibrosis. Interestingly, the deletion of *Smad2* alone did not affect adverse myocardial fibrosis, while *Smad3* seemed to be indispensable to the pressure-overload-induced fibrotic response. Moreover, cardiac dysfunction and maladaptive hypertrophy were prevented in *Tgfbr1/2* KOs, but deletion of *Smad2*, *Smad3*, *Smad2/3* did not alter the ultimate decompensation of pressure-overloaded hearts. In contrast to this observation, Russo et al. [38] found that *Smad3* deletion from activated fibroblast accelerated early systolic dysfunction after pressure overload. A difference in experimental design could explain this disparity between the cardiac phenotypes observed. Specifically, Russo et al. noticed cardiac dysfunction in *Smad3* KOs during the adaptive phase of injury, when the SMAD3-dependent matrix preservation program is critical to protect the heart from injury. Khalil et al. missed this difference as they examined the effect of gene deletion at a later time point, i.e., during the advanced stage of cardiac disease. In the case of MI, myofibroblast-specific *Smad3* deletion reduced collagen content, impaired scar organization, and increased the incidence of scar rupture and mortality [35]. In a transgenic mouse model of slow progressive genetic cardiomyopathy, myofibroblast-specific *Tgfbr2* ablation at the early stage of disease progression reduced myocardial fibrosis, alleviated cardiac hypertrophy, improved cardiac function, and extended the lifespan of the diseased mice [36]. All these studies indicate that the FB-specific TGF-β signaling response varies considerably depending on context and plays a diverse role in the pathophysiology of cardiac diseases.

### 2.2. Co-Operation between Canonical Wnt/β-Catenin and TGF-β1-SMAD3 Signaling in Fibrosis

Glycogen synthase kinase-3 (GSK-3) is a family of ubiquitously expressed serine/threonine kinases. It was first identified in 1980 for its role in regulating glycogen synthase, the rate-limiting enzyme in glycogen synthesis [93]. The GSK-3 family consists of two isoforms, α, and β. These isoforms share a 98% homology in their kinase domains but differ substantially in their N- and C-terminal sequences [94,95]. The role of the GSK-3 family of kinases in cardiomyocyte biology is well established [96,97,98,99,100,101,102]. However, in vivo studies supporting the role of the GSK-3 family of kinases in myocardial fibrosis are just beginning to emerge [26]. We employed both *Postn-Cre* and *Col1a2-Cre* to investigate the role of cardiac fibroblast (CF) GSK-3β in myocardial fibrosis [26]. We reported that the CF-specific deletion of GSK-3β lead to excessive fibrogenesis, left ventricular dysfunction, and profound scarring in the infarcted heart. Mechanistically, GSK-3β deletion in cardiac fibroblasts caused the hyperactivation of SMAD3, resulting in excessive fibrosis [26]. In stark contrast, the FB-specific deletion of GSK-3α protected against TAC-induced myocardial fibrosis and cardiac dysfunction [44]. Interestingly, GSK-3α appears to promote fibrosis via the GSK-3α-ERK-IL-11 signaling circuit. We believe that GSK-3α-mediated pro-fibrotic signaling is among the few TGF-β1/SMAD3 independent pro-fibrotic signaling cascades identified to date. Future studies are warranted in order to further validate the GSK-3α-ERK-IL-11 signaling circuit as a therapeutic target for the management of myocardial fibrosis. 

**Figure 1:**
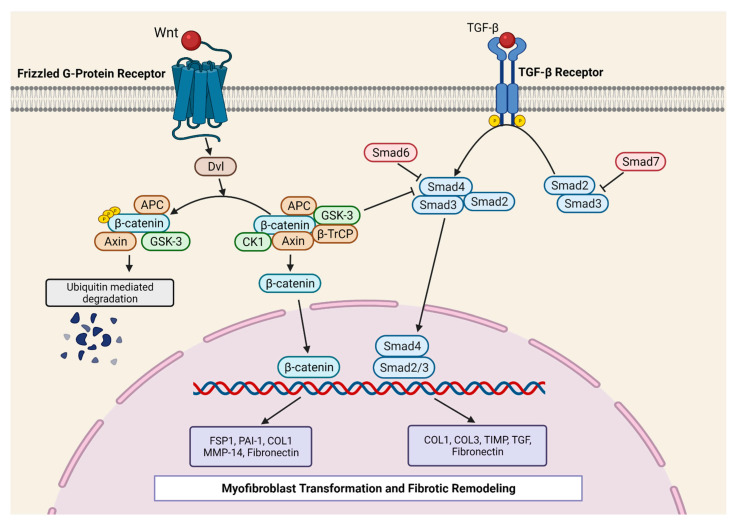
Co-operation of canonical Wnt/β-Catenin and TGF-β1-SMAD3 signaling in fibrosis. In the canonical TGF-β1-SMAD3 pathway, the binding of ligands (TGF-β) to TGF-βR leads to the phosphorylation of R-SMADs (SMAD2 and SMAD-3). Phosphorylated R-SMADs promote the association with Co-SMAD (SMAD4), resulting in nuclear translocation to mediate the pro-fibrotic gene program. The sketch shows the critical crosstalk between the canonical TGF-β1 pathway and the Wnt/β-catenin signaling. Wnt/β-catenin signaling has a dual role in pro-fibrotic signaling: (I) the direct effect of nuclear β-catenin on pro-fibrotic gene program and (II) an indirect effect through the modulation of the TGF-β1 pathway. APC = Adenomatous Polyposis Coli; β-TrCP = β-transducin repeat-containing protein CK1; Col = Collagen; Dvl = Disheveled; FSP1 = Fibroblast-specific protein 1; GSK-3 = Glycogen synthase kinase 3; MMP = Matrix metalloproteinases; PAI-1 = Plasminogen activator inhibitor-1; Smad = Contraction of Sma and Mad (Mothers against decapentaplegic); TGF-β1 = Transforming growth factor beta 1; TIMP = Tissue inhibitors of MMPs.

It is well recognized that GSK-3β plays an essential role in the Wnt/β-catenin signal transduction pathways [103]. The components and the molecular mechanism of Wnt/β-catenin signaling have been reviewed recently [104]. Briefly, in the absence of Wnt, GSK-3β participates in the destruction complex, where it phosphorylates β-catenin, leading to its ubiquitination and subsequent proteasomal degradation. However, the binding of Wnt ligands to frizzled receptors causes the disassembly of the destruction complex, thereby preventing GSK-3β mediated β-catenin phosphorylation and degradation. This leads to the stabilization and cytoplasmic accumulation of β-catenin, which further translocates into the nucleus and activates specific gene programs. Deb et al. [55] showed that acute ischemic injury induced Wnt1 expression in cardiac FBs and promoted cell proliferation in a β-catenin-dependent manner. To further confirm the biological relevance of Wnt/β-catenin signaling in adverse cardiac remodeling, they interrupted Wnt signaling by deleting β-catenin in FBs. This loss-of-function approach demonstrated minimal collagen deposition at the injury site with accelerated cardiac dysfunction and hypertrophy post-MI, suggesting that β-catenin-dependent signaling is required to maintain cardiac homeostasis in the ischemic heart. On the other hand, Xiang et al. [43] demonstrated that the loss of β-catenin in cardiac fibroblasts or myofibroblasts (*Tcf21*- or *Postn-Cre*) protects from pressure-overload-induced cardiac dysfunction and reduces interstitial fibrosis. These beneficial effects were seen despite the absence of any significant alteration in the FBs’ proliferation. However, β-catenin is known to interact with T-cell factor/lymphoid enhancer factor (TCF/LEF) gene sequences promoting the transcription of ECM genes. Nonetheless, the precise mechanism of the profibrotic role of GSK-3β-β-catenin signaling and its crosstalk with the TGF-β1 pathway is just beginning to emerge and needs further investigation [103] (Figure 1). 

### 2.3. Molecular Mechanism of p38 MAPK Mediated Pro-Fibrotic Signaling

The mitogen-activated protein kinases (MAPKs) mediate a wide range of responses to extracellular stimuli and cell functions [105,106,107]. The MAPK family consists of four sub-families: extracellular signal-regulated kinases (ERK), c-Jun N-terminal kinases (JNK/SAPK), p38, and ERK/big MAP kinase 1 (ERK5/BMK1). The role of the ERK1/2 and JNK1/2/3 signaling cascade in myocardial fibrosis has not yet been studied with in vivo mouse models. Herein, we will discuss the role of p38 MAPK in myocardial fibrosis. There are four members in the p38 family: p38α (MAPK14/SAPK2a), p38β (MAPK11/SAPK2b), p38γ (MAPK12/SAPK3), and p38δ (MAPK13/SAPK4). Out of these, the p38α and p38β isoforms are ubiquitously expressed, while p38γ and p38δ expression varies, depending on the type of tissue [108].

**Figure 2:**
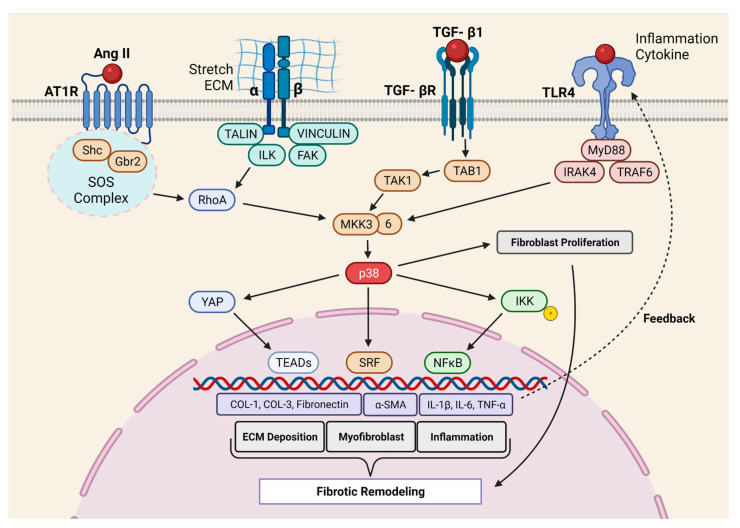
Molecular mechanism of p38 MAPK-mediated pro-fibrotic signaling. The p38 pathway is a critical positive regulator of myocardial fibrosis. At the receptor level, p38 signaling is activated by a variety of stimuli, including Ang II (AT1R), mechanical sensing (integrins), and inflammatory cytokines (TGF-βR, TLR4). These membrane-proximal events lead to the activation of MKK3/6, the specific upstream activator of the p38 MAPK kinases. Once activated, p38 crosstalk with IKK-NFkB signaling and Hippo effector YAP to mediate the pro-fibrotic (Col-1, Col-3, FN, and α-SMA) and pro-inflammatory (Il-1b, IL-6, TNF-α) gene programs. Ang II = Angiotensin II; α-SMA = Alpha-smooth muscle actin; AT1R = Angiotensin II receptor; Col = Collagen; ECM = Extracellular Matrix; FAK = Focal adhesion kinase; GBR2 = Growth factor receptor-bound protein 2; IKK = IκB kinase; IL = Interleukin; ILK = Integrin-linked kinase; IRAK4 = Interleukin 1 receptor-associated kinase 1; MKK = Mitogen-activated protein kinase kinase; MyD88 = Myeloid differentiation factor 88; NFkB = Nuclear factor κ B; RhoA = Ras homolog family member A; Shc = SH2-containing collagen-related proteins; SOS complex = Son of sevenless guanine nucleotide exchange factor; SRF = Serum response factor; TAB1 = TAK binding protein 1; TAK1 = TGF-β-activated kinase 1; TEAD = TEA domain family member; TGF-β1 = Transforming growth factor beta 1; TGF-βR = TGF-β receptor; TLR4 = Toll-like receptor 4; TNF-α = TNF, tumor necrosis factor α; TRAF6 = TNF receptor-associated factor; YAP 1 = Yes-associated protein 1.

Molkentin et al. provided the first direct evidence for the role of p38 in cardiac fibrosis [37]. They developed genetic mouse models in which p38α could be deleted from fibroblast or myofibroblast using tamoxifen-inducible *Tcf21-* and *Postn-* promoter-driven Cre recombinase, respectively. The deletion of p38α from the fibroblast prevented myofibroblast transformation and reduced fibrosis in two different mouse models of cardiac injury (IR and chronic neurohumoral-AngII stimulation). Fibroblast-specific p38α KO mice showed a higher incidence of scar rupture and 100% mortality after MI, thereby highlighting the critical role of this signaling axis in maintaining the structural integrity of the injured myocardium. Conversely, the expression of constitutively active p38α in fibroblast led to the development of cardiac fibrosis in transgenic mice, even in the absence of injury signals, further supporting the crucial role of p38α in fibrosis. In another study, Bageghni et al. [60] deleted p38α from fibroblast using tamoxifen-inducible *Col1a2-Cre-ER(T)* and observed protection against cardiac hypertrophy induced by isoproterenol (ꞵ-adrenergic receptor agonist). The authors further demonstrated that FB-p38α regulates cardiomyocyte hypertrophy in a paracrine manner through IL-6 secretion. Recently, the Davis group [30] engineered a biomimetic that recapitulates the spatial variations in collagen organization seen in ischemic hearts. Using this novel tool, the authors showed that topological disorganizations in the ECM lead to p38-dependent YAP stabilization in FBs. Indeed, YAP promotes myofibroblast transformation and myocardial fibrosis. Taken together, the p38 MAPK signaling is among the best-characterized positive regulators of fibroblast activation and myocardial fibrosis (Figure 2). 

### 2.4. GPCR-Mediated Myocardial Pro-Fibrotic Signaling

G-protein-coupled receptors (GPCRs) signaling has been extensively linked with the pathogenesis of cardiac diseases. Many conventional therapies for HF, such as beta-blockers and angiotensin-II receptor blockers (ARBs) work by targeting GPCRs. GPCRs represent the largest family of transmembrane receptors. In classical GPCR signaling, the binding of ligands induces conformational changes in the receptor, thereby activating G protein and inducing intracellular signaling cascades. The G-protein-coupled receptor kinases (GRK) are the negative regulators of GPCR signaling. Specifically, GRKs phosphorylate ligand-bound GPCRs and create a docking site for ꞵ-arrestin. This high-affinity binding of ꞵ-arrestin leads to the desensitization or downregulation of GPCRs. GRKs are a family of serine/threonine kinases. Based on tissue specificity and sequence homology, GRKs are further classified into 3 subfamilies: rhodopsin kinases (GRKs 1 and 7); β-adrenergic receptor kinases (GRKs 2 and 3); and the GRK4 subfamily (GRKs 4, 5, and 6) [109,110,111]. In the heart, the expression of GRK2 and GRK5 isoforms is predominant. A large body of research adequately supports a central role for cardiomyocyte GRKs in the pathogenesis of cardiac diseases [112,113]. However, studies investigating the FB-specific role of GRKs in cardiac diseases are limited.

**Figure 3:**
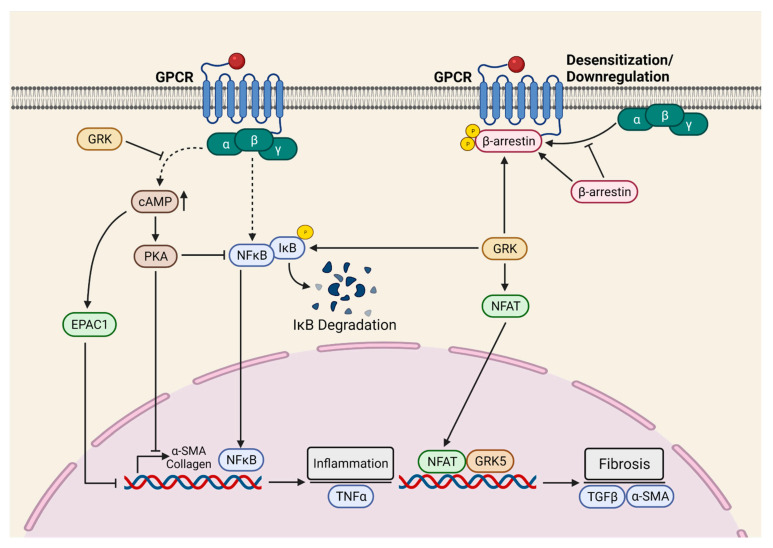
GPCR-mediated myocardial pro-fibrotic signaling. The G-protein-coupled receptor kinases (GRKs) are crucial for GPCR signaling. GRKs are the critical positive regulator of fibroblast activation and myocardial fibrosis. GRKs mediate pro-fibrotic effects by modulating cAMP levels and NF-κB signaling. Furthermore, GRKs interact with NFAT to potentiate NFAT: DNA binding, thereby inducing the transcription of NFAT-mediated fibrotic genes. α-SMA = Alpha-smooth muscle actin; cAMP = Cyclic adenosine monophosphate; EPAC = Exchange protein activated by cAMP; GRK = G-protein-coupled receptor kinases; IkB = Inhibitor of NF-κB; NFAT = Nuclear factor of activated T-cells; NFκB = Nuclear factor-kappa B; PKA = Protein kinase A; TGF-β1 = Transforming growth factor beta 1; TNF = Tumor necrosis factor.

To determine the role of cardiac fibroblast GRK2 in myocardial fibrosis, Koch et al. employed inducible fibroblast-specific GRK2 KOs [62]. Indeed, cardiac fibroblast GRK2 deletion protected against ischemia/reperfusion (I/R)-induced cardiac injury and adverse remodeling [62]. Consistently, pharmacological inhibition or the targeting of GRK2 in activated fibroblast attenuated pathological myofibroblast activation, interstitial fibrosis, and HF progression [42]. These protective effects were associated with a reduction in fibrotic and inflammatory responses in the re-perfused hearts. Mechanistically, it was proposed that GRK2 mediates pro-fibrotic effects by modulating cAMP levels in fibroblasts. Furthermore, GRK2 acts as a positive regulator of NF-κB signaling and promotes inflammatory cytokine secretion in the ischemic heart. The Koch laboratory employed two different mouse models, specifically MI and in vivo AngII infusion, to investigate the fibroblast-specific role of GRK5 in the pathogenesis of cardiac diseases. In both models, the FB-specific deletion of GRK5 prevented adverse cardiac remodeling and improved systolic function. Furthermore, the authors demonstrated that non-canonical interaction between GRK5 and NFAT potentiates NFAT: DNA binding, thereby inducing the transcription of NFAT-mediated fibrotic genes [58]. Additionally, the activation of β2-adrenergic receptors (β2AR) leads to the proliferation of cardiac proliferation and fibrosis through the Gαs/ERK1/2-dependent IL-6 secretion [114]. However, the role of β2AR in cardiac fibrosis needs further validation with conditional FB-specific mouse models in an in vivo setting. Taken together, GPCRs-mediated signaling, specifically β2AR, GRK2, and GRK5, is the critical positive regulator of myocardial fibrosis, therefore representing a novel therapeutic target for the limitation of excessive myofibroblast activation and interstitial fibrosis in the diseased heart (Figure 3). 

### 2.5. Hippo Signaling Pathway in Myocardial Fibrosis

The Hippo signaling pathway was first identified in *Drosophila* and was found to be evolutionary conserved. It controls organ size by regulating cell proliferation and apoptosis [115,116,117]. The Hippo signaling network is complex as it operates with more than 30 different components. In mammals, this signaling initiates at the mammalian Ste20-like kinases (MST1/2), which are orthologous to *Drosophila* Hippo. MST1/2 forms a complex with adaptor protein, a Sav family WW domain-containing protein 1 (SAV1) that allows the phosphorylation/activation of the large tumor suppressor 1/2 (LATS 1/2). LATS 1/2 regulates the transcriptional activities of two transcriptional co-activators, yes-associated protein (YAP) and the transcriptional co-activator with PDZ-binding motif (TAZ). When the pathway is active, LATS 1/2 phosphorylates YAP and TAZ, thereby sequestering them in the cytoplasm, eventually leading to their ubiquitin-mediated proteasomal degradation. However, the inactivation of this pathway permits the stabilization and nuclear translocation of YAP and TAZ, whereupon they interact with the TEA domain family (TEAD1-4) of transcription factors and enhance the expression of target genes [115,116,117]. Mechanotransduction plays an important role in the determining the subcellular localization of YAP/TAZ. Specifically, ECM elasticity and Rho ATPase-mediated cytoskeleton dynamics have been linked to YAP/TAZ activation [118,119]. 

**Figure 4:**
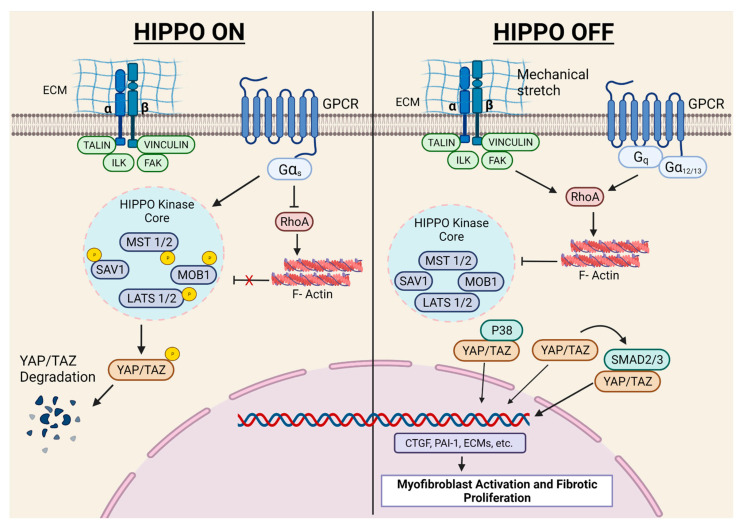
Hippo pathway and myocardial fibrosis. When Hippo signaling is on (left), the mammalian STE20-like protein kinase 1 (MST1), MST2, and the Sav family WW domain-containing protein 1 (SAV1) complex activate the large tumour suppressor homologue 1 (LATS1) and LATS2 kinases, which in turn phosphorylate and promote the degradation of the downstream effectors YAP and TAZ. When Hippo signaling is off (right), YAP and TAZ function as transcriptional co-activators and partner with different transcription factors to regulate gene transcription. Active Hippo signaling is essential to maintain fibroblasts in their inactive state (calm). However, Hippo signaling is switched off in activated fibroblasts, leading to YAP/TAZ nuclear localization for the mediation of pro-fibrotic signaling (right). CTGF = Connective tissue growth factor; ECM = Extracellular matrix; FAK = Focal adhesion kinase; Gα and Gq = G protein subunit; ILK = Integrin-linked kinase; LATS 1/2 = Large Tumor Suppressor; MOB1 = Mps One Binder 1; MST 1/2 = Mammalian Ste20-like 1 and 2; PAI-1 = Plasminogen activator inhibitor-1Roa; Smad = Contraction of Sma and Mad (Mothers against decapentaplegic); TAZ = Transcriptional co-activator with PDZ-binding motif; YAP = Yes-associated protein.

The Hippo pathway plays a critical role in cardiac development, cardiomyocyte biology, and regeneration, which has recently been elegantly reviewed [120]. Herein, we will exclusively focus on in vivo studies with FB-specific mouse models investigating the role of this signaling in fibroblast biology and fibrosis. Fransisco et al. [46] showed that YAP expression increased in FBs after MI, and FB-specific YAP ablation attenuated MI-induced cardiac dysfunction and fibrosis. The authors further demonstrated that YAP promoted myofibroblast differentiation and ECM gene expression through MRTF-A. Martin’s laboratory demonstrated that Hippo signaling promoted epicardial progenitors to fibroblast transition during embryonic development [121]. In another study, the same group conditionally deleted the Hippo pathway kinases LATS1 and LATS2 from adult mouse cardiac fibroblasts. Interestingly, the ablation of LATS1/2 from adult resting cardiac FBs caused spontaneous myofibroblast transformation, cardiac fibrosis, and systolic dysfunction, even in the absence of any cardiac insult. Moreover, the basal fibrotic response (without injury) became more severe in LATS1/2 KOs post-MI, resulting in a poor survival rate. The authors of this study employed single-cell transcriptome analysis and demonstrated that LATS1/2 are essential to the maintenance of FBs in the resting state [47]. These findings are important since there is a general belief that fibroblasts have a minimal role in resting heart physiology. Indeed, most of the research on myocardial fibrosis is limited to diseased conditions. Thus, future investigations are needed to identify the physiological role of fibroblasts in the healthy heart (Figure 4). 

## 3. Conclusions and Future Perspectives

As discussed throughout this review, the recent emergence of the conditional FB-specific mouse model revolutionized the area of cardiac fibroblast biology and myocardial fibrosis research. As a result, the last decade was productive, leading to a paradigm shift towards the idea that fibrosis is not merely a secondary effect of developing pathology and that fibroblasts are not just ECM-producing cells. In fact, numerous studies showed that FB-specific gene targeting can lead to a robust cardiac phenotype, including cardiomyocyte hypertrophy. Indeed, the animals studied demonstrated intact gene expression in all other cells, including the cardiomyocytes. Thus, the last decade saw remarkable progress in our understanding of cardiac fibroblasts’ role in myocardial pathophysiology. There was reasonable success on the mechanism front as well; in addition to the historical profibrotic canonical TGF-β1 pathway, numerous new pro- and anti-fibrotic pathways were identified. However, although substantial progress has been made, it will require a great deal of effort to transform these early bench discoveries into much-needed anti-fibrotic therapies for patients. Specifically, most of the work performed is focused on linear pathways; it is conceivable that these newly identified pathways operate through multiple crosstalk. These signaling circuits and missing links are yet to be established. An upcoming area of enormous potential is the cellular crosstalk among cardiac cells (e.g., fibroblasts to cardiomyocytes) and the circulation (immune cells). The area of fibroblast crosstalk with the immune system and its role in myocardial injury, healing, regeneration, and fibrosis is gaining a lot of interest and is currently under intense investigation by multiple groups. Furthermore, the interplay of fibroblast and inflammation is proposed to play a critical role in the pathogenesis of the comparatively understudied HFpEF syndrome. Regrettably, due to the lack of well-optimized animal models of HFpEF, the progress in this exciting area has been slow and is only just starting to gain some momentum. In the same vein, cardiomyocytes and fibroblast cellular crosstalk has been noted in multiple settings. However, the precise mechanism is not known and is currently under intense investigation by numerous groups. Another emerging area is fibroblast heterogeneity within organs and across various tissues. This line of research with single-cell multi-omics and advanced genomics technologies will be critical to the identification of commonalities and heterogeneity among fibrotic diseases across organs and could play a crucial role in drug repurposing. Indeed, we have recently reported the potential of repurposing Nintedanib (an FDA-approved kinase inhibitor for pulmonary fibrosis) to combat myocardial fibrosis, pathological cardiac remodeling, and dysfunction [122]. The repurposing of authorized anti-fibrotic agents is certainly a “low hanging fruit,” and this line of investigation should be prioritized. Moreover, we anticipate that various fibroblast subpopulations may have a distinct role in the repair and remodeling of the injured heart. Efforts from multiple laboratories with the single-cell RNAseq approach have paved the way towards the identification of the specific markers of different fibroblast subpopulations. We speculate that this knowledge base will help to create future FB-specific mouse models with the ability to target particular FB subpopulations. Finally, it is clear that a better understanding of the profibrotic signaling network may provide a promising new therapeutic target for managing myocardial fibrosis. An effective translation of these new findings will need rigorous verification in human tissues and human tissue-based culture systems, such as pluripotent cell-derived organoids (human tissue chips). There is great optimism that with these newly optimized models and identified pathways, the area of myocardial fibrosis research is set to see another round of growth and productivity. We anticipate that these efforts will enable new approaches to the prevention, treatment, and, hopefully, even the reversal of myocardial fibrosis in diseased hearts. 

## Figures and Tables

**Table 1:** List of proteins/markers that have been targeted in order to create mouse lines for FB-specific genetic manipulation and lineage tracing.

Proteins/Markers	Biological Role	Expression in FB States	Expression in Other Cells	References
Periostin	ECM protein	Developmental stage, activated FBs	Epicardium	[9,18,21,22,23,24,25,26,27,28,29,30,31,32,33,34,35,36,37,38,39,40,41,42,43,44]
TCF21	Transcription factor	Resting FB, downregulate in activated FBs	Epicardium	[9,20,22,23,31,37,41,43,44,45,46,47,48,49]
α-SMA	Cytoskeletal protein	Activated FBs	Pericytes, VSMC, Epicardium	[6,9,12,25,50]
Collagen I, III	ECM protein	Resting and activated FBs	Pericytes, VSMC, Endothelial cells, Cardiomyocytes	[12,20,22,25,26,27,45,51,52,53,54,55,56,57,58,59,60,61,62]
CD90	Cell-cell interaction	Resting and activated FBs	Pericytes, VSMC, immune cells, Endothelial cells	[12,27,45,51,52,53]
DDR2	Cell-ECM interaction	Resting FBs	Epicardium	[22,25,63,64,65]
FSP1	Calcium binding protein	Resting and activated FBs	Pericytes, VSMC, Immune cells, Endothelial cells	[9,18,19,22,23,25,66,67]
Fibronectin	ECM protein	Resting and activated FBs	Endothelial cells	[22,68,69,70]
PDGFRα	Tyrosine kinase receptor	Resting and activated FBs	Cardiac progenitor cells	[9,12,22,25,71,72,73]
Stem cells antigen-1	Stem cell antigen	Resting and activated FBs	Cardiac progenitor cells	[22,74,75,76]
Vimentin	Cytoskeletal protein	Resting and activated FBs	Pericytes, VSMC, Endothelial cells	[22,25,77,78,79,80]

**Table 2:** Studies carried out with FB-specific, genetically manipulated mouse models.

Target Gene	Promoter Used for Cre Expression	Major Findings	References
*Tgfbr1/2, Smad2, Smad3*	*Postn*	FB-specific deletion of *Tgfbr1/2* or *Smad3*, but not *Smad2*, markedly reduced fibrosis in pressure-overloaded mouse hearts as well as fibrosis mediated by heart-specific, latency-resistant TGF-β mutant transgene.	[33]
*Smad3*	*Postn*	In pressure-overloaded hearts, the protective actions of the myofibroblasts were mediated through *Smad3*-dependent matrix-preserving program	[38]
*Smad3*	*Postn*	FB-specific *Smad3* loss impaired scar remodeling and increased the incidence of late rupture post-MI	[35]
*Tgfbr2*	*Postn*	*Tgfbr2* ablation in the myofibroblast prevented fibrosis and cardiac dysfunction in mouse model of cMyBP-C-induced cardiomyopathy	[36]
*Gsk3b*	*Postn*	FB-specific deletion of GSK-3β lead to the hyperactivation of SMAD-3, resulting in excessive fibrotic remodeling and cardiac dysfunction after myocardial infarction.	[26]
*Gsk3a*	*Tcf21* and *Postn*	In pressure-overloaded hearts, FB-specific GSK-3α mediated pro-fibrotic effects through an ERK-IL-11 circuit that operated independently of TGF-β/SMAD3 signaling	[44]
*Ctnb1*	*Tcf21* and *Postn*	Loss of β-catenin in fibroblasts attenuated pressure-overload-induced cardiac fibrosis	[43]
*p38*	*Tcf21* and *Postn*	FB-specific deletion of p38 attenuated myofibroblasts transformation and fibrosis. Conversely, transgenic mice expressing constitutively active p38 in FB specific manner develops fibrosis in multiple organs.	[37]
*p38*	*Postn*	Spatial variations in collagen organization regulated cardiac fibroblast phenotype through the mechanical activation of p38-YAP-TEAD signaling	[30]
*Grk2*	*Postn*	Ablation of GRK2 in activated fibroblasts significantly reduced myofibroblast transformation and fibrosis and showed cardiovascular protection post-I/R injury	[42]
*Lats1/2*	*Tcf21*	FB-specific deletion of *Lats1* and *Lats2* initiated a self-perpetuating fibrotic response in the uninjured adult heart that was exacerbated by MI	[47]
*Yap*	*Tcf21*	FB-specific deletion of YAP prevented MI-induced cardiac fibrosis and dysfunction through MRTF-A inhibition.	[46]
*Htr2b*	*Tcf21* and *Postn*	Deletion of 5-HT_2B_ receptor signaling in fibroblast prevented border zone expansion and improved microstructural remodeling after MI	[41]
*Hsp47*	*Postn*	Myofibroblast-specific ablation of *Hsp47* blocked fibrosis in mouse models of pressure overload, MI and, muscular dystrophy	[34]
*Sox9*	*Postn*	FB-specific deletion of *Sox9* ameliorated MI-induced left ventricular dysfunction, inflammation, and myocardial scarring	[39]
*Kcnk2*	*Tcf21*	FB-specific deletion of TREK1 prevented pressure-overload-induced deterioration in cardiac function	[48]
*Rock2*	*Postn*	Deletion of ROCK2 in fibroblast attenuated cardiac hypertrophy, fibrosis, and diastolic dysfunction in mice subjected to chronic Ang-II infusion	[40]
*Fn1*	*Tcf21*	FB-specific fibronectin gene ablation ameliorated adverse cardiac remodeling and fibrosis post I/R	[49]
*Prkaa1*	*Postn*	AMPKα1 deletion in myofibroblasts exacerbated post-MI adverse fibrotic remodeling	[32]
*Sptbn4*	*Postn*	FB-specific deletion of βIV-spectrin aggravated Ang-II induced fibrosis and cardiac dysfunction.	[84]
*Pmca4*	*Postn*	FB-deletion of PMCA4 reduced TAC-induced hypertrophy and cardiac dysfunction	[24]
*Mbnl1*	*Tcf21* and *Postn*	Deletion of MBNL1 impaired the fibrotic phase of wound healing in mouse models of MI.	[31]
*Klf5*	*Postn*	FB–specific KLF5 deletion ameliorated TAC-induced cardiac hypertrophy and fibrosis	[27]
*Postn*	*Tcf21* and *Postn*	Ablation periostin expressing FBs reduced collagen production and scar formation after MI.	[9]
*Postn*	*Postn*	Ablation of periostin expressing FBs reduced fibrosis and improved cardiac function in mice subjected to chronic Ang-II infusion as well as in mice after MI	[21]

## Data Availability

Not applicable.
